# Dietary effect of pomegranate seed oil rich in 9cis, 11trans, 13cis conjugated linolenic acid on lipid metabolism in obese, hyperlipidemic OLETF Rats

**DOI:** 10.1186/1476-511X-3-24

**Published:** 2004-11-09

**Authors:** Keisuke Arao, Yu-Ming Wang, Nao Inoue, Junichi Hirata, Jae-Young Cha, Koji Nagao, Teruyoshi Yanagita

**Affiliations:** 1 Department of Applied Biological Sciences, Saga University, Saga 840-8502, Japan

## Abstract

Conjugated fatty acid, the general term of positional and geometric isomers of polyunsaturated fatty acids with conjugated double bonds, has attracted considerable attention because of its potentially beneficial biological effects. In the present study, dietary effect of pomegranate seed oil rich in punicic acid (9*cis*, 11*trans*, 13*cis*-conjugated linolenic acid; 9c, 11t, 13c-CLNA) on lipid metabolism was investigated in obese, hyperlipidemic Otsuka Long-Evans Tokushima Fatty (OLETF) rats. After 2 weeks feeding period, OLETF rats revealed obesity and hyperlipidemia compared with their progenitor LETO rats. Feeding of the diet supplemented with 9% safflower oil and 1% pomegranate seed oil (9c, 11t, 13c-CLNA diet) did not affect abdominal white adipose tissue weights and serum lipid levels compared with the diet supplemented with 10% safflower oil (control diet) in OLETF rats. However, the accumulated hepatic triacylglycerol was markedly decreased by 9c, 11t, 13c-CLNA diet in OLETF rats. Activities of hepatic enzymes related to fatty acid synthesis and fatty acid β-oxidation were not altered by 9c, 11t, 13c-CLNA diet. Levels of monounsaturated fatty acid (MUFA), major storage form of fatty acid, in serum triacylglycerol were markedly higher in obese, hyperlipidemic OLETF rats than in lean LETO rats. In addition, 9c, 11t, 13c-CLNA diet significantly decreased MUFA levels in OLETF rats. This is the first study showing that 9c, 11t, 13c-CLNA suppresses delta-9 desaturation in vivo, and we suggest that the alleviation of hepatic triacylglycerol accumulation by 9c, 11t, 13c-CLNA diet was, at least in part, attributable to the suppression of delta-9 desaturation in OLETF rats.

## Background

Conjugated fatty acid (CFA) is the general term of positional and geometric isomers of polyunsaturated fatty acids with conjugated double bonds. It has been reported that conjugated linoleic acid (CLA), the CFA form of linoleic acid, has favorable physiological effects, such as anti-atherosclerosis, anti-obesity, anti-tumor, and anti-hypertension [1-9]. There are also other types of CFA in some plant seed oils. Punicic acid (9*cis*, 11*trans*, 13*cis*-conjugated linolenic acid; 9c, 11t, 13c-CLNA) is contained about 72% in pomegranate seed oil [10]. α-Eleostearic acid (9*cis*, 11*trans*, 13*trans*-CLNA) is contained in bitter gourd oil and tung seed oil about 60% and 70%, respectively [10,11]. Catalpa seed oil also contains catalpic acid (9*trans*, 11*trans*, 13*cis*-CLNA) about 31% and pot marigold seed oil contains calendic acid (8*trans*, 10*trans*, 12*cis*-CLNA) about 33% [10]. There are some studies showing that mixtures of CLNA isomers, prepared by alkaline isomerization of α-linolenic acid or plant seed oil, have some physiological functions including body fat reduction and anti-tumor activity [12,13]. In addition, purified α-eleostearic acid (9c, 11t, 13t-CLNA) and α-eleostearic acid rich bitter gourd seed oil also reveal anti-carcinogenesis in vitro and in vivo [10,11,14,15]. However, there are few studies evaluated the physiological function of punicic acid (9c, 11t, 13c-CLNA) [10,16]. Previously, we reported the hypolipidemic effect of purified punicic acid in human liver derived HepG2 cells [17].

In the present study, we investigated the effects of pomegranate seed oil rich in 9c, 11t, 13c-CLNA on lipid metabolism in Otsuka Long-Evans Tokushima fatty (OLETF) rats. OLETF rats develop a syndrome with multiple metabolic and hormonal disorders that shares many features with human obesity [18-21]. OLETF rats have hyperphagia, because they lack receptors for cholecystokinin, and become obese, developing hyperlipidemia, diabetes, and hypertension. To clarify the physiological function of 9c, 11t, 13c-CLNA, we measured hepatic enzyme activities in relation to lipid metabolism and fatty acid composition in plasma of these obese, hyperlipidemic rats.

## Results and Discussion

In comparison with their progenitor Long-Evans Tokushima Otsuka (LETO) rats, OLETF rats had increased body weight gain with enhanced food intake during 2 weeks feeding period (Table 1). In OLETF rats, food intake was not different between the groups. There was also no significant difference between groups in the relative liver weights of LETO and OLETF rats. Food efficiency, however, was higher in 9c, 11t, 13c-CLNA group than in other two groups. Chin et al. previously reported that CLA is a growth factor for rats as shown by enhanced weight gain and improved feed efficiency [22]. Thus, we consider that 9c, 11t, 13c-CLNA may have some growth promotional function.

**Table 1:** Effect of 9c, 11t, 13c-CLNA on body weight, relative liver weight, food intake, and food efficiency

	**LETO**	**OLETF**	
		
		**Control**	**9c, 11t, 13c-CLNA**
Body weight (g)			
Initial	223 ± 3^a^	266 ± 12^b^	265 ± 8^b^
Final	282 ± 5^a^	357 ± 15^b^	369 ± 11^b^
Gain	59.4 ± 2.7^a^	91.3 ± 3.6^b^	104 ± 6^b^
Relative liver weight (g/100 g BW)	3.12 ± 0.09	3.40 ± 0.11	3.35 ± 0.06
Food intake (g)	17.8 ± 0.4^a^	25.8 ± 1.1^b^	26.2 ± 1.1^b^
Food efficiency (g BW gain/g intake)	25.7 ± 1.0^a^	27.3 ± 0.6^b^	30.4 ± 1.1^b^

^a,b^Different superscript letters show significant difference at *P *< 0.05.

**Figure 1:**
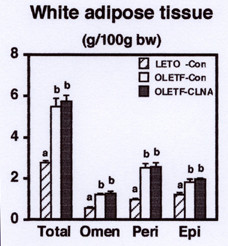
Effect of 9c, 11t, 13c-CLNA on abdominal white adipose tissue weight in LETO and OLETF rats. Rats were fed a control or 9c, 11t, 13c-CLNA diet for 2 weeks. Values are expressed as mean ± SE for 6 rats. ^a,b^Different letters show significant differences at *P *< 0.05. Omen, omental; Peri, perirenal; Epi, epididymal.

The effect of dietary 9c, 11t, 13c-CLNA on the accumulation of abdominal white adipose tissue (WAT) was investigated (Figure 1). After 2 weeks feeding period, OLETF rats developed marked abdominal obesity. Compared with LETO rats, the control diet increased perirenal, epididymal, and omental WAT weights of OLETF rats to 2.6-, 1.5-, and 2.1-fold, respectively. There was no significant effect of 9c, 11t, 13c-CLNA on the accumulation of abdominal WAT in OLETF rats. However, 2 weeks feeding of the diet supplemented with 5% pomegranate seed oil resulted in a significant reduction of omental WAT weight (by 27%) compared with the feeding of control diet in OLETF rats (unpublished data). These results suggested that 2 weeks feeding of 1% pomegranate seed oil diet might not be enough to reveal anti-obese effect of 9c, 11t, 13c-CLNA.

**Figure 2:**
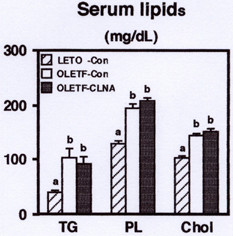
Effect of 9c, 11t, 13c-CLNA on serum lipid levels in LETO and OLETF rats. Rats were fed a control or 9c, 11t, 13c-CLNA diet for 2 weeks. Values are expressed as mean ± SE for 6 rats. ^a,b^Different letters show significant differences at *P *< 0.05. TG, triacylglycerol; PL, phospholipids; Chol, cholesterol.

After the 2 weeks feeding period, OLETF rats revealed hyperlipidemia. Serum triacylglycerol, phospholipids, and cholesterol levels of OLETF rats fed the control diet were significantly higher than those of LETO rats fed the control diet (Figure 2). However, feeding of 9c, 11t, 13c-CLNA did not affect to serum lipid levels in OLETF rats. Although the present results showing that dietary 1% pomegranate seed oil rich in 9c, 11t, 13c-CLNA could not alleviate hyperlipidemia in OLETF rats, our previous report indicated that purified 9c, 11t, 13c-CLNA suppressed the secretion of apolipoprotein B100 from human liver derived HepG2 cells [17]. Further studies are needed to elucidate the effect of purified 9c, 11t, 13c-CLNA on the pathogenesis of hyperlipidemia in OLETF rats.

**Figure 3:**
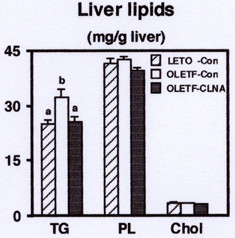
Effect of 9c, 11t, 13c-CLNA on hepatic lipid levels in LETO and OLETF rats. Rats were fed a control or 9c, 11t, 13c-CLNA diet for 2 weeks. Values are expressed as mean ± SE for 6 rats. ^a,b^Different letters show significant differences at *P *< 0.05. TG, triacylglycerol; PL, phospholipids; Chol, cholesterol.

Next, we investigated the effect of dietary 9c, 11t, 13c-CLNA on the distribution of lipids to the liver. There was no significant difference in relative liver weight between control and 9c, 11t, 13c-CLNA group in OLETF rats. Previous reports indicated that CLA feeding resulted in the development of hepatomegaly and fatty liver in mice [23-25], and a mixture of CLNA also induced hepatic lipid accumulation in rat [13]. In the present study, the triacylglycerol concentration in OLETF rats was significantly higher than that in LETO rats, and the triacylglycerol accumulation in the liver of OLETF rats was markedly alleviated by the 9c, 11t, 13c-CLNA diet (Figure 3). There was no significant difference in liver phospholipids and cholesterol levels among groups in LETO and OLETF rats. These results suggest that 9c, 11t, 13c-CLNA has a preventive effect against the triacylglycerol accumulation in the liver.

**Figure 4:**
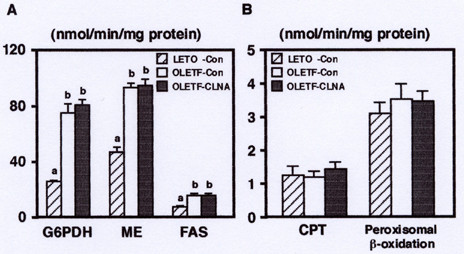
Effect of 9c, 11t, 13c-CLNA on activities of enzymes related to lipid metabolism, (A) G6PDH, ME, FAS (B) CPT, peroxisomal β-oxidation, in the liver of LETO and OLETF rats. Rats were fed a control or 9c, 11t, 13c-CLNA diet for 2 weeks. Values are expressed as mean ± SE for 6 rats. ^a,b^Different letters show significant differences at *P *< 0.05.

To further investigate the regulation of hepatic lipid metabolism, we analyzed the effect of dietary 9c, 11t, 13c-CLNA on the activities of enzymes related to fatty acid synthesis and fatty acid β-oxidation. As shown in Figure 4A, the activities of glucose-6-phosphate dehydrogenase (G6PDH) and malic enzyme (ME), key enzymes of NADPH production, and fatty acid synthase (FAS), a key enzyme of fatty acid synthesis, were markedly increased in OLETF rats fed the control diet compared with LETO rats. There was no significant effect of dietary 9c, 11t, 13c-CLNA on these enzyme activities in OLETF rats. The activities of carnitine palmitoyltransferase (CPT), a key enzyme of fatty acid β-oxidation, and peroxisomal β-oxidation were not different between OLETF and LETO rats, and 9c, 11t, 13c-CLNA diet did not affect on these activities in OLETF rats (Figure 4B). Koba et al. previously reported that a mixture of CLNA isomers, prepared by alkaline isomerization, enhanced hepatic mitochondrial and peroxisomal β-oxidation compared with linoleic acid, α-linolenic acid, and CLA [13]. Thus, we consider that the effect of 9c, 11t, 13c-CLNA on the fatty acid β-oxidation is weak compared with those of other CLNA isomers. In addition, the alleviation of hepatic triacylglycerol accumulation by 9c, 11t, 13c-CLNA could not be attributed to the regulation of enzyme activities related to the fatty acid synthesis and fatty acid β-oxidation.

To gain insight into the effect of dietary 9c, 11t, 13c-CLNA on lipid metabolism, we analyzed fatty acid composition in serum triacylglycerol. As shown in Table 2, saturated fatty acid (SFA) levels were lower and monounsaturated fatty acid (MUFA) levels were higher in OLETF rats fed the control diet than those in LETO rats. Feeding of 9c, 11t, 13c-CLNA significantly reduced MUFA levels in plasma triacylglycerol of OLETF rats. It has been recognized that MUFAs are the major fatty acid form in fat depots [26]. Alterations in the ratio of SFA to MUFA have been implicated in various disease states including cardiovascular disease, obesity, and diabetes [27-29]. Therefore, the ratio of SFA to MUFA is of physiological importance in normal and disease states. A key enzyme involved in the cellular synthesis of MUFA from SFA is the membrane-bound stearoyl-CoA desaturase (SCD), which inserts a cis-double bond in the delta-9 position of fatty acid substrates. Previous reports indicated that 10t, 12c-CLA, an active isomer of anti-obese effect of CLA, suppresses delta-9 desaturation and SCD activity in vitro and in vivo [30-32]. In the present study, the index of delta-9 desaturation, ratio of oleic acid (18:1) versus stearic acid (18:0), was higher in obese, hyperlipidemic OLETF rats compared with in lean LETO rats, and it was significantly decreased by dietary 9c, 11t, 13c-CLNA in OLETF rats. As far as we know, this is the first study showing that 9c, 11t, 13c-CLNA also suppresses delta-9 desaturation in vivo. We suggest that the alleviation of hepatic triacylglycerol accumulation by dietary 9c, 11t, 13c-CLNA was, at least in part, attributable to the suppression of delta-9 desaturation in OLETF rats.

**Table 2:** Effect of 9c, 11t, 13c-CLNA on fatty acid composition in serum triacylglycerol.

	**LETO**	**OLETF**	
		
		**Control**	**9c, 11t, 13c-CLNA**
	%		
14:0	2.28 ± 0.23^a^	1.05 ± 0.15^b^	1.10 ± 0.13^b^
16:0	36.8 ± 0.7^a^	28.1 ± 0.7^b^	41.2 ± 2.7^a^
16:1	0.492 ± 0.066^a^	3.83 ± 0.25^b^	2.39 ± 0.20^c^
18:0	5.58 ± 0.63^a^	3.29 ± 0.26^b^	4.04 ± 0.32^b^
18:1	11.7 ± 0.7^a^	24.5 ± 1.4^b^	20.4 ± 1.4^c^
18:2	35.5 ± 0.7^a^	33.2 ± 0.9^a^	27.8 ± 1.4^b^
20:4	7.74 ± 0.76^a^	4.80 ± 0.40^b^	3.12 ± 0.44^c^
Desaturation index			
Δ9 desaturation	2.18 ± 0.24^a^	7.73 ± 0.83^b^	5.34 ± 0.75^c^
Δ6 desaturation	0.219 ± 0.024^a^	0.144 ± 0.009^b^	0.110 ± 0.011^b^

^a,b,c^Different superscript letters show significant difference at *P *< 0.05.

## Conclusions

Dietary pomegranate seed oil rich in 9c, 11t, 13c-CLNA alleviates hepatic triacylglycerol accumulation in obese, hyperlipidemic OLETF rats. The mechanism of this effect could not be attributed to the regulation of enzyme activity related to fatty acid synthesis and fatty acid β-oxidation. However, suppression of delta-9 desaturation by dietary 9c, 11t, 13c-CLNA may be, at least in part, involved this effect.

## Materials and Methods

### Animals and diets

All aspects of the experiment were conducted according to the guidelines provided by the ethical committee of experimental animal care at Saga University. Five weeks old male OLETF rats and LETO rats, the progenitor of OLETF rats, were provided by Tokusima Research Institute (Otsuka Pharmaceutical, Tokushima, Japan). Rats were housed individually in metal cages in temperature-controlled room (24°C) under a 12-hour light/dark cycle. After a 1-week adaptation period, OLETF rats were assigned to two groups (six rats each) that were fed with a semisynthetic diet supplemented with 10% safflower oil (the control group) or a semisynthetic diet supplemented with 9% safflower oil and 1% pomegranate seed oil rich in 9cis, 11trans, 13cis-CLNA (the 9c, 11t, 13c-CLNA group). LETO rats were fed the same diet as the OLETF rats in the control group. The pomegranate seed oil rich in 9c, 11t, 13c-CLNA (69.0%) was prepared by Kaneka Co. (Hyogo, Japan). The semisynthetic diet were prepared according to recommendations of the AIN-76 [33] and contained (in weight %): casein, 20; fat, 10; cornstarch, 15; vitamin mixture (AIN-76™), 1; mineral mixture (AIN-76™), 3.5; DL-methionine, 0.3; choline bitartrate, 0.2; cellulose, 5; and sucrose, 45. The rats received different diets for 2 weeks and were killed by aortic exsanguinations under diethyl ether anesthesia. Liver and abdominal (perirenal, epididymal, and omental) WATs were also excised for analysis.

### Analysis of lipids

Serum was separated by centrifuging the blood. Triacylglycerol, cholesterol, and phospholipids in serum were measured using enzyme assay kits from Wako Pure Chemicals (Tokyo, Japan). Liver lipids were extracted and purified according to the method of Folch et al [34]. The concentrations of triacylglycerol, cholesterol, and phospholipids were measured according to the methods of Fletcher [35], Sperry and Webb [36], and Bartlett [37]. Measurement of fatty acid composition in plasma was carried out as previously described [38,39].

### Preparation of liver subcellular fractions

A piece of liver was homogenized in 6 volumes of a 0.25 M sucrose solution that contained 1 mM EDTA in a 10 mM tris Tris-HCL buffer (pH 7.4). Fractions of mitochondria, microsomes, and cytosol were obtained as previously described[40]. The protein concentration was determined according to the method of Lowry et al [41], with bovine serum albumin used as the standard.

### Assays of enzyme activity

The enzyme activities of ME (EC 1.1.1.40) [42], G6PDH (EC1.1.1.49) [43], FAS (EC 2.3.1.85) [44] in the liver cytosol fraction, mitochondrial CPT (EC2.3.1.23) [45] and peroxisomal β-oxidation [46] were determined as described.

### Statistical analysis

All values are expressed as means ± SE. Data were analyzed by one-way ANOVA, and all differences were inspected by Duncan's new multiple-range test [47]. Differences were considered to be significant at *P*<0.05.

## List of abbreviations

CFA, conjugated fatty acid; CLA, conjugated linoleic acid; CLNA, conjugated linolenic acid; OLETF rat, Otsuka Long-Evans Tokushima fatty rat; LETO rat, Long-Evans Tokushima Otsuka rat; WAT, white adipose tissue; G6PDH, glucose-6-phosphate dehydrogenase; ME, malic enzyme; FAS, fatty acid synthase; CPT, carnitine palmitoyltransferase; SFA, saturated fatty acid; MUFA, monounsaturated fatty acid; SCD, stearoyl-CoA desaturase
